# Thinking About Behavior: Perspective on Meta-Behavior in Education

**DOI:** 10.3389/fpsyg.2021.727116

**Published:** 2021-08-12

**Authors:** Muhammad Syawal Amran, Suriana Mohd Zain, Khairul Azhar Jamaludin, Shahlan Surat

**Affiliations:** ^1^Faculty of Education, National University of Malaysia, Bangi, Malaysia; ^2^Sekolah Menengah Kampung Jawa, Klang, Malaysia

**Keywords:** meta-behavior, thinking, metacognition, conceptual framework, perspectives

## Abstract

Studies on metacognitive skills have started in the past 30 years and cover various fields, including education. In general, metacognitive skills refer to awareness and monitoring cognitive processes and their ability to sharpen the mind enhancement process. However, much attention has been given to metacognition alone and less focusing on its manifestation in behaviors. Thus, this study aims to conceptualize how metacognitive concepts can be adapted in the context of behaviors. In achieving this, an in-depth analysis of relevant behavioral theories and metacognitive models was conducted. The proposed conceptual framework, named the meta-behavior framework, underscores the importance of the thinking process before an individual engages in action. Undoubtfully, this skill is vital in influencing an individual to plan, monitor and evaluate his/her actions in daily life. In short, the proposed framework is essential in expanding the current knowledge terrain on psychology, which specifically provides a new perspective in understanding how the thinking process determines behavior.

## Thinking About Behavior

Over the past three decades, research on metacognitive abilities grew exponentially after Flavell (1979) coined the term metacognitive referring to an understanding of cognition. This term was widely used, and it has piqued the interest of scholars, leading to the development of a complex research topic in educational science. Metacognitive skills refer to an individual’s understanding and control over thought processes. Indeed, the ability to control metacognitive processes aids in the discovery of methods to implement an effective strategy to achieve optimal results, particularly in decision making and strategy (Koriat, 2015; Smortchkova and Shea, 2020).

Metacognitive skills play an essential role in cognitive activities and processes, such as communication, language, perception, and attention retention. Metacognitive aids in the organization of cognitive activities before executing a decision or action (Schraw and Dennison, 1994; Pelton, 2014; Simons et al., 2020). Furthermore, metacognitive skills aid in the monitoring of knowledge to achieve the goal of cognition. The concept of metacognition does not simply imply that one is aware and believes in organizing and carrying out cognitive activities. Instead, it is more important to understand how cognition occurs (Pelton, 2014; Doyle and Hourihan, 2016; Simons et al., 2020). Thus, metacognition necessitates specific knowledge and regulating one’s thinking processes toward activities, operations, and cognitive strategy.

Wilson and Bai (2010) established that metacognitive abilities influence behavior, decision-making capability, and moral behavior. They also described how metacognitive processes guide thinking in selecting the appropriate strategy for generating problem-solving ideas. However, focusing exclusively on metacognition is insufficient in the absence of its behavioral manifestation and thus demonstrates a knowledge gap regarding the processing of stimuli before their manifestation as observable behaviors. Additionally, limited discussion of metacognition is evident in describing how the mental processes involved in this process would determine the locus of behavioral control (Abdullah et al., 2019).

Indeed, the cognitive approach emphasizes the mental processes necessary to interpret, learn, comprehend, and regulate thought than the behavioral approach. Its core value is in the scientific activities or observable responses of an organism. Nonetheless, neither approach is regarded as distinct from the other. Instead, it forms an interaction that can be translated from the cognitive processes into behaviors. Rahman and Abdullah (2013) conducted research and introduced the concept of meta-behavior, which combines the concepts of metacognition (as proposed by Flavell (1979), cognitive regulation, and behaviors (as described in research by Schraw and Dennison, 1994) to demonstrate that a particular behavior requires planning, monitoring, and evaluation (metacognitive strategy) prior to manifesting observable behaviors.

In short, meta-behavioral skill is a mental process that occurs before an individual engages in action. This ability is critical because it affects the actions required to plan, control, and evaluate daily activities, enabling an individual to think before acting. The expanded definition of meta-behavior depicts the interaction between cognition and behavior. In comparison to metacognition, the term meta-behavior has received little attention, and thus, it should be put forward in research. It is particularly important in understanding how thinking processes influence behavior.

## Meta Behavioral From Theoretical Perspectives

Rahman and Abdullah (2013) cautioned that an analysis of metacognitive and behavioral theories should be systematically conducted in understanding its complex relationship. Thus, a cross-interaction between metacognitive and behavioral theories is used as a foundation to propose the meta-behavior framework, demonstrating that demonstrated action requires planning, monitoring, and evaluation at the execution level, which refers to the metacognitive strategy before it manifests observable behaviors.

According to Flavell’s metacognitive theory (1979), the concept of knowledge and metacognitive experience shape behavioral knowledge. This process includes analyzing the concept, facts, idea, and knowledge about action and how and what behavior is appropriate in executing the ideas. Flavell (1979) further elucidated that this process incorporates retention and acquisition of knowledge on appropriate behavioral planning. Similarly, Schraw and Dennison’s (1994) metacognition model proposed that knowledge and regulation about cognition are combined to form behavioral concepts that contribute to the meta-behavior strategy. This strategy entails mental activities that assist an individual in planning, monitoring, and evaluating an action before it being manifested physically (Oxley, 2018; Simons et al., 2020).

According to Ajzen’s (1991) theory of planned behavior, three major factors shape behaviors: attitudes (belief about other’s attitudes toward a particular behavior), subjective norms (belief which is in approval of other people’s view or belief), and perceived behavior control (one’s belief on his/her ability to perform a specific behavior). This means that these three factors play an important role in shaping behavior. Ajzen (1991) firmly believed that individuals would make rational and reasoned judgments to engage in a certain behavior by evaluating the available information, which resonates with Flavell’s (1979) claim. Therefore, individuals are likely to engage with rational and reasoned judgments behavior if: (a) they believe that the behavior will lead to particular outcomes which they value, (b) the behavior is in line with normative belief, and they feel they are capable and have opportunities and adequate resources to perform the behavior (Conner and Norman, 1994; Ajzen, 2012; Ajzen and Sheikh, 2016; Burn and Roszkowska, 2016).

In fact, Freud’s psychoanalytic theory (1991) described that all features and behaviors are the results of psychic motivators that stimulate behaviors and are based on the id, ego, and superego. The ego is an executive component that strives to make a reasoned decision, whereas the id refers to the urge to indulge in delight without evaluating the implications. On the other hand, the superego gathers moral values and functions according to moral principles to govern the id and ego in the decision-making process. These three components are hypothetical constructions that are supposed to exist in people’s minds, and each has its role and interacts with one another to guide an individual to reach a decision. In this process, an individual will involve in planning, monitoring, and evaluation to choose a course of action (Freud, 1991).

On the other hand, prosocial theory explains how moral development influences an individual’s behavior. Moral behavior is shaped by highlighting the transition of internalized moral values (Eisenberg and Shell, 1986). Moral behavior is a result from an individual’s internalization of moral values that become a guiding principle in his or her life. This is critical to ensuring that the behavior portrayed is morally acceptable, especially when rules and laws are involved and the moral evaluation requires a systematic mental ability to stimulate an individual’s behavior and decision-making. In a recent work by Drigas and Mitsea (2020), they proposed the eight pillars model of metacognition which includes: (a) deep theoretical knowledge of cognition, (b) operational knowledge of cognitive function, (c) self-monitoring, (d) self-regulation, (e) physical, emotional, and cognitive function adaptation, (f) internal and external recognition of an object, (g) discrimination of function and facilitation of our work or target, and (h) Mnemosyne. Clearly, their work similarly highlights the importance of self-observation, regulation and recognition to change to metacognition, and the connections between metacognition, self-consciousness and behavior. It was established that these components are vital to elaborate how to achieve mindfulness which is not only restricted to learning. Based on the eight pillars, Mitsea and Drigas (2019) have proposed metacognitive learning strategies that are essential not only self-regulated learning but also for self-regulated behavior.

**FIGURE 1:**
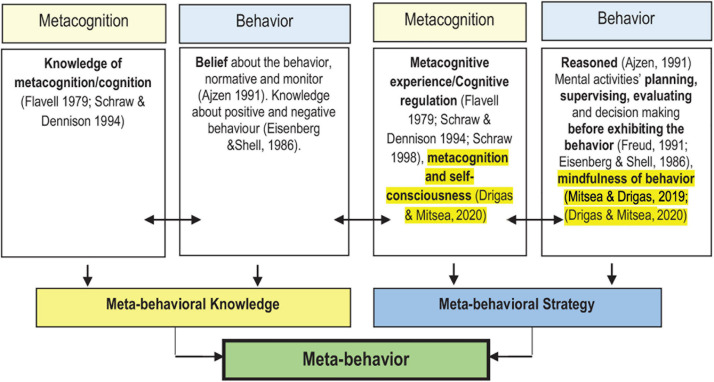
Theoretical perspective that indicates the formation of meta-behavior adapted from Abdullah and Rahman (2018).

In a nutshell, the concept of metacognitive skills (Flavell, 1979; Schraw and Dennison, 1994) integrates behavioral concepts (as exemplified in Ajzen’s theory of planned behavior, Freud’s psychoanalysis, Piaget’s model of development, Kohlberg’s theory of moral development, and Eisenberg and Shell’s model of prosocial behavior). Metacognitive skills are visible not only in mental activities but also in behavior. The cross-interaction of behavioral theory and metacognition is depicted in Figure 1 below.

## A Conceptual Framework of Meta Behavior

Meta-behavioral skills are the thinking processes that occur before an individual acts. This ability is critical for influencing someone’s behavior to plan, monitor, and evaluate his or her daily actions. The fundamental element in this framework is to elaborate on the mental processes in response to a received stimulus. This is because the process of interaction between metacognitive and behavioral is the result of the stimuli of experience that the individual has gone through. In other words, the past experience will shape the knowledge to make choices to more adaptive behaviors and make decisions to behaviors (Frith, 2012; Veenman, 2012). The integration of several metacognitive models, such as those by Flavell (1979) and Schraw and Dennison (1994), has resulted in developing a meta-behavioral framework that comprises two components: meta-knowledge and meta-behavioral strategy. Integrating these two central concepts creates a mind-execution capability that interacts with behavior before its representation.

According to Abdullah and Rahman (2018), meta-knowledge is defined as knowledge about concepts, facts, an idea about action, and knowledge about how to perform the action and in which situation, the action is deemed appropriate. Additionally, it refers to the memory process and what one knows about behavior before its manifestation through action. Meta-behavior is constructed based on three components: declarative knowledge, procedural knowledge, and conditional knowledge. As implied by the functions, the metaphysical foundation is an idea, belief, or awareness that describes the procedure and suitability for knowledge to occur.

**FIGURE 2:**
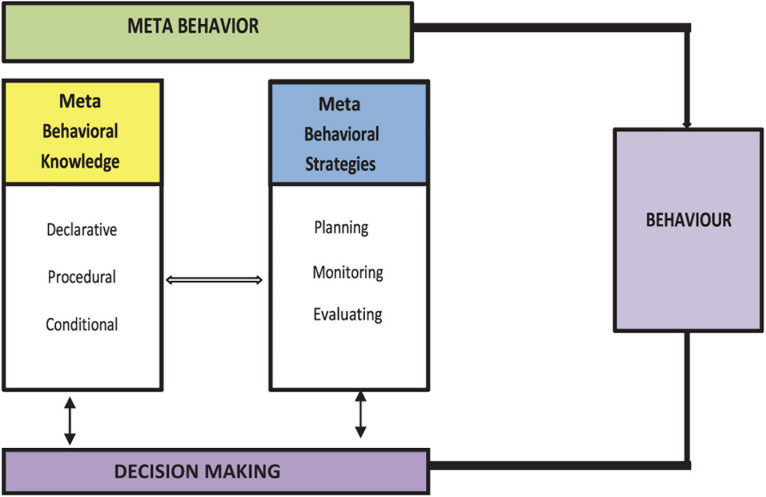
Conceptual meta-behavior skills framework adapted from Abdullah and Rahman (2018).

Additionally, the meta-behavioral strategy incorporates meta-behavioral skills, which involve mental activities that assist students in planning, monitoring, and evaluating their actions before they manifested as observable behaviors. The meta-behavioral strategy is composed of three subcomponents: planning, monitoring, and evaluation. Abdullah and Rahman (2018), added that integrating the two pivotal concepts of metacognition and behavior serves as the mind’s execution power—interacting with one another before the behavior manifesting during the learning process. Based on these premises, it is relevant to conclude that meta-behavioral skills can serve as a predictor of an individual’s actions as their interaction influences the manifestation of behaviors and decision-making. In short, the meta-behavior skills framework (as illustrated in Figure 2) explains the role of each meta-behavior element in influencing an action.

## Conclusion

Undeniably, the term meta-behavior has received little attention in previous research in comparison to metacognition. This article outlines a foundation in expanding the theory and its application in future research. This is important, especially in determining what and how metacognition affects behaviors. Undeniably, meta-behavioral skills are pivotal as they enable individuals to predict and plan a course of action to prepare for a crisis internally before deciding on a sound course of action. In other words, individuals will be less inclined toward compulsive behaviors and deception or bias from external sources when their thinking processes are activated.

Additionally, meta-behavioral skills necessitate further investigation as a novel dimension for comprehending behavioral issues. The disjunction between organizing and interacting elements of meta-knowledge and meta-behavior has been interpreted as the source of distorted internal locus of control in behavioral problems. Individuals who are aware of these skills may be more optimistic, open, and capable of empowering themselves and willingly improving their weaknesses when confronted with conflict. Thus, the greater the meta-behavioral abilities, the more effective the behaviors manifested (Flavell, 1979; Abdullah and Rahman, 2018).

Additionally, the discussed skills are viewed as a self-ability that enables an individual to holistically reach his or her full potential. This is because the internal locus of control serves as the anchor for one’s strength. Individuals who practice these skills will develop a consistent metacognitive approach that helps developing sound behavior. In conclusion, a great deal of information and positive experience will be stored in a reservoir of self. As with life achievement, the reservoir of experience results from the harmonious integration of continuous meta-behavioral skills (Flavell, 1979). Indirectly, it shapes an exemplary personality and inspires others. In short, meta-behavioral skill is a novel perspective that should be widely explored in the future as the findings might expand the existing theory and practice, especially in the field of psychology.

## Author Contributions

MA and KJ: conception and design of the work. SZ and SS: drafting the work and revising the final approval of the work. All authors contributed to the article and approved the submitted version.

## Conflict of Interest

The authors declare that the research was conducted in the absence of any commercial or financial relationships that could be construed as a potential conflict of interest.

## Publisher’s Note

All claims expressed in this article are solely those of the authors and do not necessarily represent those of their affiliated organizations, or those of the publisher, the editors and the reviewers. Any product that may be evaluated in this article, or claim that may be made by its manufacturer, is not guaranteed or endorsed by the publisher.
